# Dbx1-Derived Pyramidal Neurons Are Generated Locally in the Developing Murine Neocortex

**DOI:** 10.3389/fnins.2018.00792

**Published:** 2018-10-31

**Authors:** Eneritz Rueda-Alaña, Isabel Martínez-Garay, Juan Manuel Encinas, Zoltán Molnár, Fernando García-Moreno

**Affiliations:** ^1^Achucarro Basque Center for Neuroscience, Edificio Sede del Parque Científico de la UPV/EHU, Leioa, Spain; ^2^Department of Physiology, Anatomy and Genetics, University of Oxford, Oxford, United Kingdom; ^3^School of Biosciences, Cardiff University, Cardiff, United Kingdom; ^4^Ikerbasque – Basque Foundation for Science, María Díaz de Haro, Bilbao, Spain; ^5^Department of Neurosciences, University of the Basque Country (UPV/EHU), Leioa, Spain

**Keywords:** tangential migration, ventral pallium, ventral migratory stream, claustrum, olfactory cortex, Nurr1

## Abstract

The neocortex (NCx) generates at the dorsal region of the pallium in the forebrain. Several adjacent structures also contribute with neurons to NCx. Ventral pallium (VP) is considered to generate several populations of neurons that arrive through tangential migration to the NCx. Amongst them are the Cajal-Retzius cells and some transient pyramidal neurons. However, the specific site and timing of generation, trajectory of migration and actual contribution to the pyramidal population remains elusive. Here, we investigate the spatio-temporal origin of neuronal populations from VP in an *in vivo* model, using a transposase mediated in utero electroporation method in embryonic mouse. From E11 to E14 cells born at the lateral corner of the neocortical neuroepithelium including the VP migrated ventro-laterally to settle all areas of the ventral telencephalon. Specifically, neurons migrated into amygdala (Ag), olfactory cortices, and claustrum (Cl). However, we found no evidence for any neurons migrating tangentially toward the NCx, regardless the antero-posterior level and developmental time of the electroporation. Our results challenge the described ventral-pallial origin of the transient pyramidal neuron population. In order to find the exact origin of cortical neurons that were previously Dbx1-fate mapped we used the promoter region of the murine *Dbx1* locus to selectively target Dbx1-expressing progenitors and label their lineage. We found these progenitors in low numbers in all pallial areas, and not only in the ventral pallial ventricular zone. Our findings on the local cortical origin of the Dbx1-derived pyramidal neurons reconcile the observation of Dbx1-derived neurons in the cortex without evidence of dorsal tangential migration from VP and provide a new framework for the origin of the transient Dbx1-derived pyramidal neuron population. We conclude that these neurons are born locally within the dorsal pallial neuroepithelium.

## Introduction

Neocortex (NCx) relies on a delicate balance of developmental events, such as neurogenesis, neuronal migration, axogenesis, and cellular death within the dorsal pallium (DP). Several neuronal populations contribute to the formation of cortical laminae. Most pyramidal excitatory neurons are born locally in the germinative zones (GZs) of the DP and reach the CP by radial migration (Nadarajah and Parnavelas, 2002). In addition, tangential migration provides a source for cortical neuronal diversity. Neurons born at the medial, lateral, and caudal ganglionic eminences in the subpallium (SP) migrate tangentially toward the neocortical neuroepithelium and differentiate into GABAergic interneurons (Cooper, 2013). These are the two main neuronal populations in the mature NCx. In addition, several other populations arrive to the DP by tangential migration form various different pallial sources and orchestrate cortical development by leading neurogenesis, circuit formation, and laminar formation (Barber and Pierani, 2015; Garcia-Moreno et al., 2018).

There is a transient pyramidal neuron population that belongs to the lineage of the Dbx1-expressing progenitors (Teissier et al., 2010, 2012). These neurons are believed to originate at the ventral pallial (VP) subventricular zone (SVZ), and are marked by the strong localized expression of the homeobox transcription factor Dbx1 (Medina et al., 2004; Bielle et al., 2005). These glutamatergic neurons colonize the entire CP and disappear during the first postnatal week (Teissier et al., 2012; Barber and Pierani, 2015). Deletion of Dbx1 results in the reduction of cortical neuronal numbers, therefore it is proposed that these transient neurons derived from a Dbx1-expressing progenitor (from now on *Dbx1-derived neurons*) promote cortical neurogenesis (Teissier et al., 2012). However, the actual contributions of the VP to this particular population and to cortical development in general are not clear due to contradictory experimental evidence (Garcia-Moreno et al., 2008; Ceci et al., 2012; Puelles et al., 2015b). Previous genetic fate mapping was performed by a combination of Dbx1-Cre and Dbx1-LacZ lineage analyses (Teissier et al., 2010; Gelman et al., 2011). These studies emphasize that the VP is the only pallial region that expresses *Dbx1* in the GZ, as previously described by *in situ* hybridization (Medina et al., 2004; Bielle et al., 2005). These genetic lineage time-course tracings revealed a population of cortical pyramidal neurons that must have derived from Dbx1-expressing progenitors. Since VP is considered to be the only pallial Dbx1 expressing region, it has been suggested that these neurons have to originate from here. Other studies using the same genetic tools arrived to the same conclusions (Gelman et al., 2011; Teissier et al., 2012). Conversely, several lines of evidence challenge this view. Whole embryo culture for short-term lineage tracing during considerable periods of neurogenesis (E10 to E13) did not reveal a dorsally migrating population from the germinative VP (Garcia-Moreno et al., 2008; Ceci et al., 2012; Frade-Pérez et al., 2017). In addition, other indirect genetic fate mapping analyses also failed to describe a dorsal component from specific populations of the corticostriatal border (Pattabiraman et al., 2014). However, no long-term *in vivo* experiments have been performed to describe the full neuronal lineage generated in the area, regardless of selective expression of Dbx1.

In this study we investigated the source of migration originating at the lateral corner of the cortical neuroepithelium that included the Dbx1 expressing VP region from E11 to E14, spanning the time of the birth of the Dbx1-derived cortical transient pyramidal neurons. We employed an *in vivo* full lineage-tracing assay based on transposase-mediated *in utero* electroporation. With this method, we labeled and studied every cell generated in the murine ventral and lateral pallia at different embryogenesis stages, regardless of the genetic expression of selective markers. We found no tangential migration generated from this boundary area independently of either the antero-posterior level or the time when electroporation was performed. In the light of these findings we aimed to determine where the previously described Dbx1-derived cortical pyramidal neurons are actually originated. We performed focal Dbx1-fate mapping by targeted electroporation of plasmids selective for Dbx1 activity. We found that a small and scattered population of neocortical dorsal pallial progenitors expresses enough Dbx1 transcripts to trigger the expression of reporter labeling. Therefore, we conclude that the Dbx1-derived transient pyramidal neuron population is generated locally from Dbx1 expressing local cortical progenitors in the DP and not from the ventral pallium (VP), from where we never observed dorsal tangential migration to cortex during the timeframe studied (E11–14).

## Materials and Methods

### Animals

All animal experiments were approved by a local ethical review committee and conducted in accordance with personal and project licenses under the UK Animals (Scientific Procedures) Act (1986) and the Spanish Government (Royal Decree 1201/2005 and 53/2013; Law 32/107). Adult C57BL/6 mice were obtained from a local breeding colony at the University of Oxford [based on the Harlan (United Kingdom) strain]. These were maintained on a 12/12-h light/dark cycle (7 AM, lights on) and provided with *ad libitum* access to food and water. The day when vaginal plug was detected was referred to as E0.

### In Utero Electroporation

Transfection by electroporation of embryonic neural progenitors was performed as described previously (Garcia-Moreno et al., 2010; Martinez-Garay et al., 2016). Briefly, E11–14 pregnant mice were anesthetized by inhalation of isoflurane administered in conjunction with 100% oxygen. After midline laparotomy, the uterine horns were exposed out of the abdominal cavity and constantly warmed and hydrated with pre-warmed sterile saline. The heads of embryos were *trans*-illuminated and injected with a glass electrode to fill up the lateral ventricle (LV) specifically. Each embryo was injected with a volume less than 1 μl comprising the mixture of plasmids. The embryos were then electroporated. Forebrain ventricular zone cells were transfected by means of a BTX Electroporator ECM 830 (Harvard Apparatus). For the electroporation we used five pulses (50 ms) discharging a 500-μF capacitor charged to 35–45 V with a sequencing power supply. The voltage pulse was discharged using a pair of platinum round plates (3 or 5 mm in diameter). Buprenorhpine (vetergesic) was administered to the pregnant mice prior to surgery (0.05 mg/kg) and the injected embryos were examined at different embryonic or postnatal stages.

The main purpose of our research is to investigate the possible source of tangential migrations originating from the lateral corner of the cortical neuroepithelium with particular interest to the Dbx1 expressing VP. For this purpose, our electroporation method for labeling neural progenitors from selected sectors of the neuroepithelium is likely the most powerful methodology. With this method, a group of progenitors is labeled and the derived cells can migrate inside the intact whole brain *in vivo*, rather than in a confined plane of a section. This method can reveal a direct relationship between the cells’ origin in the VZ and their fate within the tissue days later. Our electroporation technique is able to show (1) whether the derived cells migrate away from their site of generation, (2) whether the migratory route of the derived neurons is radial or tangential, (3) what is the distribution of the neurons, and (4) what are their characteristics. However, electroporation also have some limitations. Even if the parameters used the same, the number and exact location of transfected progenitors shows considerable variability from one animal to another.

The transposable system employed by us offers two notable improvements for the study of cellular migration. First, as it labels stem progenitors permanently, these are always detectable. Therefore we can define the telencephalic identity of the specific ventricular sector where the electroporation was performed. And secondly, since the whole progeny inherits the genomic label from the progenitor, cell populations generated late after electroporation are still detectable, as opposed to traditional non-transposable electroporation where the electroporated DNA does not get incorporated to the progenitors genome. Moreover, our selected methodology enables the observation of native three-dimensional cellular migratory trajectories in the whole intact live brain.

### Plasmids

Most of the plasmid constructs employed in this study were employed in our previous study and have been described in detail (Garcia-Moreno and Molnár, 2015). The concentration of different plasmids was kept constant among the different experiments. Labeling constructs (pPB-CAG-EYFP and pPB-CAG-STOP-mCherry) were transfected at a final concentration ranging between 200 and 500 ng/μl; the transposase enzyme expressing-construct (mPB) was consistently transfected at 300 ng/μl. Cre expressing construct under Dbx1 enhancer (pDbx1-Cre) was electroporated at 100 ng/μl. Cre expression in the pDbx1-Cre vector is driven by a 3.2 Kb sequence located 2.7 Kb upstream of the first murine *Dbx1* exon, as described in Lu et al. (1996). This sequence contains the forebrain *cis*-acting regulatory elements of the *Dbx1* gene, as represented by the 3 kb-hspLacZ construct in Figure 1B in Lu et al. (1996), and was amplified from genomic DNA using primers DbxP-1F-SalI (5′-ACATGTCGACTGTGTTTATGCGAGCGTATGCC-3′ and DbxP-1R-XhoI (5′-TATCGACTCGAGGCCGCCATTGAAAGAACAAAAT-3′). The PCR product was cloned into pGSX-Cre using SalI and XhoI. The Hsp68 minimal promoter, amplified from genomic DNA with primers Hsp68P-F-XhoI (5′-TATGCACTCG-AGGAGCCCCTACGAGCAGGGAG-3′) and Hsp68P-R-EcoRI (5′-AGGCGAA-TTCTCTGGGGAAGGCTGGTCCTG-3′) was then added downstream of the *Dbx1* enhancer using EcoRI and XhoI. We confirmed that Hsp68 minimal promoter does not show expressing activity itself when isolated from an enhancer.

### Tissue Processing

Embryonic murine brains were fixed by immersion in 4% paraformaldehyde (PFA, diluted in phosphate buffered saline 0.1 M – PBS, pH 7.3). Brains were transferred to PBS 24h after fixation. All brains were sectioned in the coronal plane at 50–70 μm thickness in a vibrating microtome (Leica VT1000S).

### Immunohistochemistry

Single and double immunohistochemical reactions were performed as described previously (Garcia-Moreno et al., 2012) using the following primary antibodies: Rabbit antibody to EGFP (Molecular Probes, A11122, 1:1000), mouse antibody to EGFP (Abcam, ab1218, 1:1000), chick antibody to EGFP (Aves GFP 1020, 1:10000), rabbit anti Nurr1 (Santa Cruz, sc-991, 1:400), rabbit antibody to Dbx1 (1:200; kind gift by Prof. Nakagawa, Univ. Minessota, United States), rabbit antibody to Tbr1 (Chemicon, AB9616, 1:1,000), rat antibody to Ctip2 (Abcam, ab18465, 1:500), rabbit antibody to dsRed2 (Takara, 632475, 1:1000), and rabbit antibody to Pax6 (Covance, PRB-278P, 1:200). Dbx1 immunostaining required antigen retrieval with citrate acid.

For secondary antibodies (all 1:1000), we used Alexa 568 goat antibody to rabbit IgG (Molecular Probes, A11011), Alexa 647 goat antibody to rabbit IgG (Molecular Probes, A21245), Alexa 488 goat antibody to rabbit IgG (Molecular Probes, A11034), Alexa 488 goat antibody to mouse IgG (Molecular Probes, A11001), Alexa 568 antibody to mouse IgG (Molecular Probes, A11004), Alexa 568 goat antibody to rat IgG (Molecular Probes, A11077), and Alexa 488 goat antibody to chicken (Invitrogen, A11039).

### Imaging and Analysis

Images were captured using a Zeiss LSM 710 confocal microscope (Carl Zeiss Microimaging). Similar image parameters (laser power, gain, pinhole, and wavelengths) were maintained for images from each brain and adjusted for new specimens as required. The employed fluorophores were DAPI, EGFP, Alexa 488, mCherry, Alexa 568, and Alexa 647. In selected cases, Z-stacks were taken individually for each channel and then collapsed to get maximum intensity projections. For panoramic views of big brain sections, tile-scan images were composed. Images were adjusted and analyzed using ImageJ (Image Analysis in Java, NIH) and Adobe Photoshop CS6 (Adobe Systems Inc.).

Images were taken from a minimum of three animals successfully electroporated in each pallial region (specific numbers for each experimental paradigm are included into the relevant figure legends). The lack of migratory cells arriving to DP from the VP corner source did not need to be quantified, as there were no cells migrating to DP in over 20 animals studied and performed during the *in vivo* period studied (E11 to E18).

The proportion of CP neurons and remaining progenitors at the GZ derived from local DP Dbx1-expressing progenitors was counted on single confocal planes. A minimum of three representative sections were used to estimate the average number of cells per animal. The data from different animals were compared by two-way ANOVA.

## Results

### Neuronal Derivatives of the Murine Ventral Pallium

**FIGURE 1:**
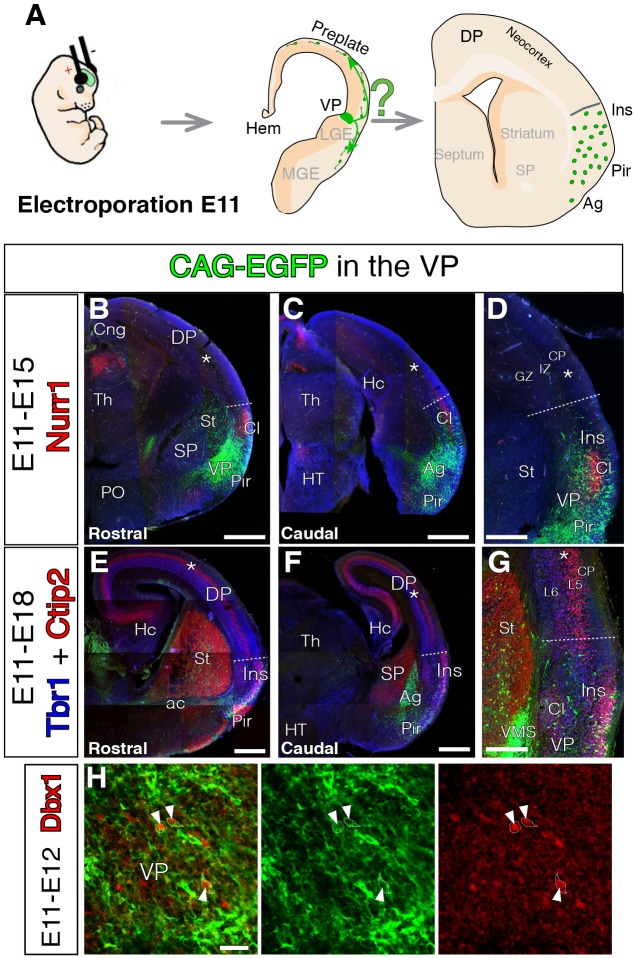
Lack of VP-derived early tangential migration to the murine neocortex (NCx). For all coronal sections, medial at the left. **(A)** Scheme depicting the experimental paradigm. E11 mouse embryos were electroporated at the ventral pallium (VP) with CAG-EGFP (green). The lineage of the electroporation was revealed at different embryonic stages, E15 or E18. **(B–G)** GFP cells derived from the lateral telencephalic wall (comprising SP, VP, and LP), electroporated at E11 and analyzed at E15 co-immunostained with Nurr1 in red (**B–D**, *n* = 3), or E18 co-immunostained with Tbr1 in blue and Ctip2 in red (**E–G**, *n* = 3). Nurr1 staining at the claustrum (Cl) clearly delineated the LP boundaries. The GFP labeled cells did not arrive to the DP at any stages of neurogenesis. Cells only settled the radial derivatives of the electroporated region in Cl, amygdala (Ag), pyriform cortex (Pir), and insular cortex (Ins). One GFP cell was observed as an exception **(E)** in the germinative zone (GZ) of the medial pallium, without connection to all the other GFP labeled cells. **(D,G)** Dashed lines mark the dorsal limit of the area occupied by the labeled cells between lns and DP. **(H)** Short-term electroporation and co-immunostaining for Dbx1 showing the electroporated area comprise VP GZs (*n* = 3) and white arrowheads depict Dbx1 immunoreactive (red) cells which were also labeled with EGFP (green). DAPI counterstain in blue in **(B–D)**. Scale bars represent 500 μm; 250 μm; and 25 μm in **(B–H)**. For nomenclature, please refer to Abbreviations list.

**FIGURE 2:**
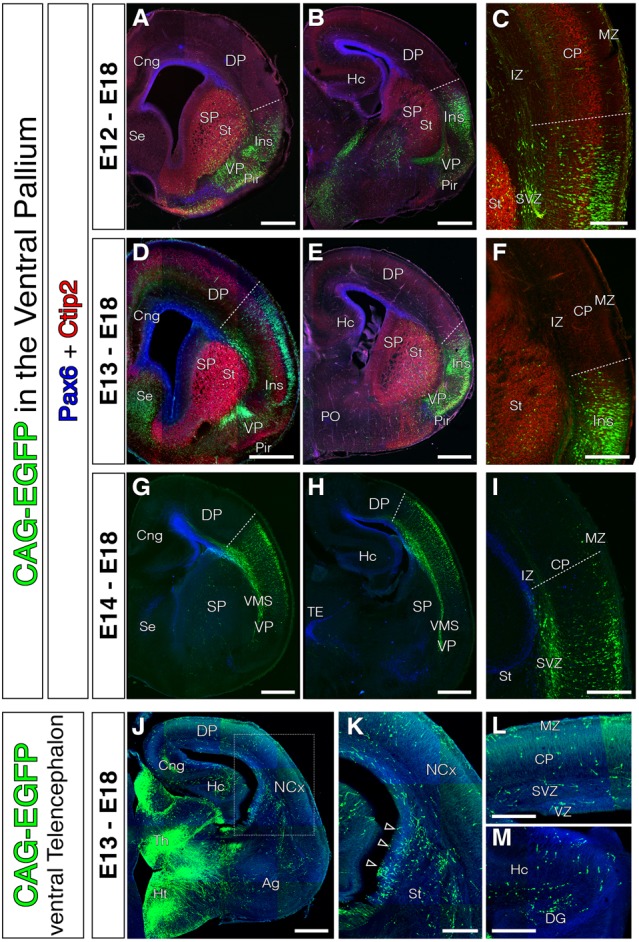
Absence of tangential migration from VP midgestational progenitors to the murine NCx. In all coronal sections, medial is at the left. **(A–I)** GFP cells derived from the lateral telencephalic wall (comprising SP, VP, LP and in occasions the most lateral DP), electroporated at E12 (**A–C**, *n* = 4), E13 (**D–F**, *n* = 3), or E14 (**G–I**, *n* = 4). We observed no cells that migrated into the DP by the end of neurogenesis (E18); all cells settled within the radial derivatives of the electroporated region (lns, Cl, VP, Pir, VMS, and Ag). Pax6 (blue) and Ctip2 (red) label telencephalic landmarks such as pallial-subpallial boundary, St respectively. **(C,F)** Dashed lines mark the dorsal limit of the area occupied by the labeled cells along the DP. Very exceptional cells were located slightly beyond this limit, but were not considered tangential cells given their proximity to the electroporation area and their radial morphology – lns boundary. **(J–L)** Electroporations at E13 on other telencephalic regions (including the ventral SP) labeled large populations of tangential migratory interneurons. **(K)** High power view of the region indicated with rectangle in **J**. **(L–M)** High power views from the dorsal NCx and hippocampus (Hc) showing the horizontally oriented tangential interneurons. DAPI counterstain in blue in **(J–M)**. Scale bars represent 500 μm and 250 μm in **(A–M)**.

Evidence from experiments using direct lineage tracing techniques revealed the extracortical origins of the glutamatergic tangential migrations for both Cajal-Retzius (Garcia-Moreno et al., 2007; Imayoshi et al., 2008) and subplate neurons (Garcia-Moreno et al., 2008; Pedraza et al., 2014). However, these techniques have not been directly applied to prove the VP origin of mammalian transient pyramidal neurons expressing Dbx1 (Teissier et al., 2010). The ventral pallial origin of these neurons is based on reporter gene expression in transgenic mouse models. In our first experiments we wanted to identify the exact location within the mouse VP from which tangentially migrating neurons are generated using *in vivo* lineage tracing methods (Puelles et al., 2015b). Therefore, we pursued the VP germinal zone during a specific neurogenic period in mouse. We electroporated EGFP expression vectors at E11 at several levels of the rostro-caudal extension of the VP (Figure 1), at the time when VP-derived cells are reported to be generated and believed to start their migration toward the NCx (Teissier et al., 2010). Electroporated brains were analyzed 4–7 days later, at E15 (Figures 1B–D) and E18 (Figures 1E–G), respectively. In order to ensure the entire germinative VP region was labeled, we analyzed brains in which the telencephalic regions flanking the VP, i.e., SP and lateral pallium (LP) were also transfected. Accordingly, electroporated brains showed EGFP+ cells in a number of different regions: (1) the striatum (St) and olfactory tubercle (derived from the striatal SP); (2) piriform cortex (Pir), endopiriform nucleus, amygdala (Ag) and ventropallial migratory stream (from the VP); and (3) lateral cerebral cortex, including claustrum (Cl) and insular cortex (Ins) (derived from LP) (Figures 1B–G). Nurr1 staining at the Cl clearly delineated the VP to LP boundary (Puelles et al., 2015a; Figures 1B–D). If cells were seen migrating to the NCx in our experiments, we could not discriminate the GZ origin of those cells. To our surprise, neither at E15 nor at E18 did we find EGFP+ cells in neocortical areas dorsal to the focus of the electroporation (Figures 1B–G). The same lack of tangential invasion of the NCx was seen in E18 brains electroporated at E12 (Figures 2A–C), E13 (Figures 2D–F), or E14 (Figures 2G–I). Thus, results were consistent in our experiments, regardless of the age at which electroporations were performed, the rostro-caudal region of VP transfected or the other pallial areas transfected (Figures 1, 2). To confirm that these results are not due to failure in labeling VP progenitors, we performed short-term tracing experiments and found that electroporated cells also expressed the VP marker Dbx1 (Figure 1H). In addition, electroporation of subpallial regions clearly labeled interneurons migrating from the ganglionic eminences into the cortex (Figures 2J–L), demonstrating the ability of the electroporation technique to capture tangentially migrating populations. Interneurons were never labeled on VP-exclusive electroporations, as the migration of these neurons does not occur in contact with the lateral ventricular surface.

### *Dbx1*-Expressing Progenitors in the Mammalian DP

**FIGURE 3:**
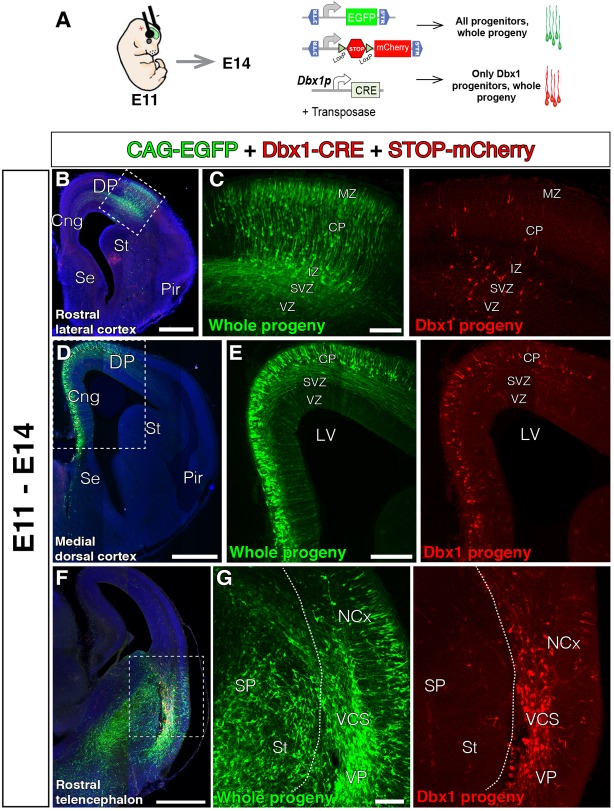
The lineage of early local DP Dbx1-expressing progenitors. In all coronal sections, medial at the left. DAPI counterstain in blue. **(A)** Electroporation paradigm at E11 (*n* = 4). The DNA cocktail simultaneously labels the lineage of any progenitors (green) and of Dbx1-expressing progenitors (red). The location of the labeled cells is analyzed and documented 3 days later. **(B–E)** Dbx1-expressing progenitors generate neurons that migrate radially to the cortical plate (CP), both at the DP-derived NCx (**B**, power view in **C**) and the MP-derived cingulate cortex (**D**, power view in **E**). Dbx1-derived neurons represented a small fraction of the total electroporated cells. **(F,G)** Power views from the lateral telencephalon. Some dorsal pallial cells are derived from Dbx1-expressing progenitors (red). At the subpallium (SP), many cells were electroporated (EGFP, green in **F,G)**, but no cells expressed the red protein, suggesting Dbx1 enhancer is selectively active in scattered pallial progenitors. Dashed line marks the pallial-subpallial boundary. Scale bars represent 500 μm; 200 μm; and 100 μm in **(B–G)**.

**FIGURE 4:**
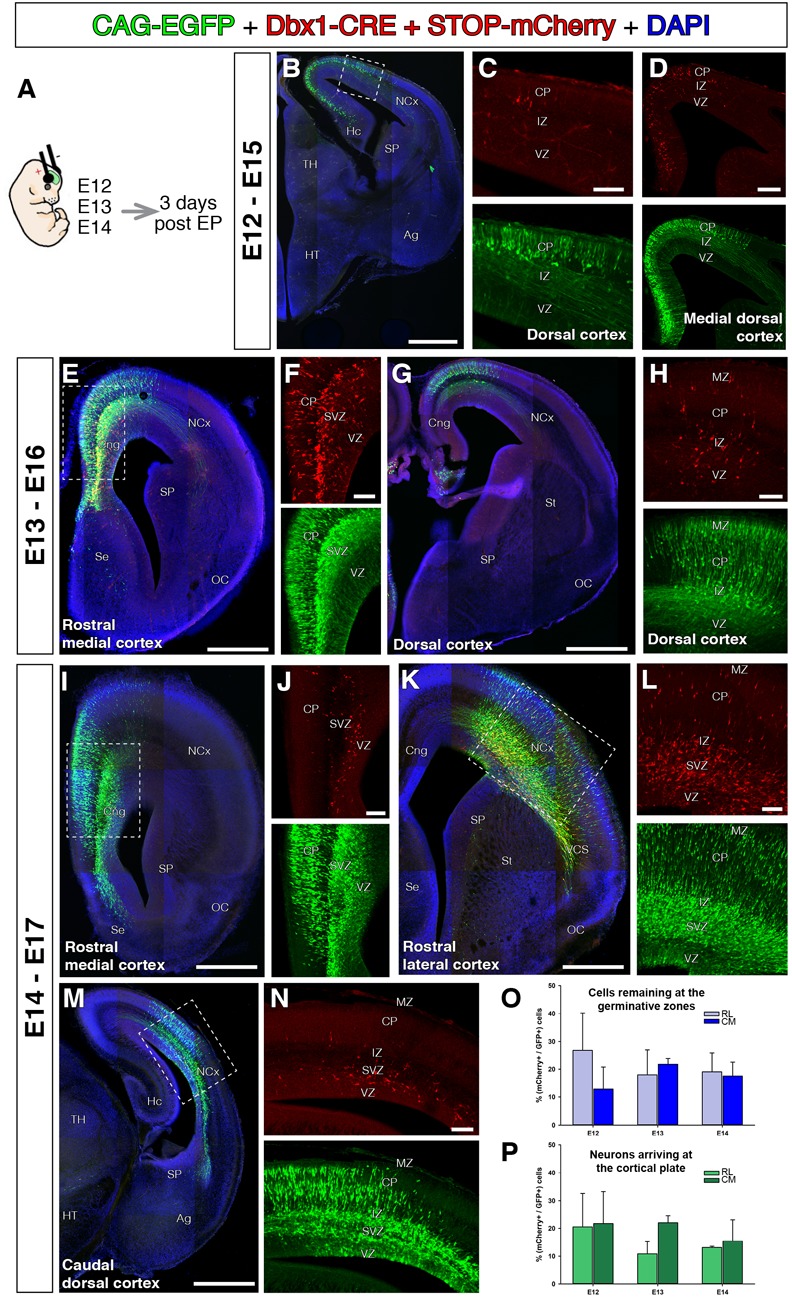
Dbx1-expressing progenitors throughout the pallium and embryonic stages. In all coronal sections, medial at the left. DAPI counterstain in blue. **(A)** Animals were electroporated following the same paradigm as in Figure 3 at different embryonic stages, E12 (**B–D**, *n* = 3), E13 (**E–H**, *n* = 2), and E14 (**I–N**, *n* = 4). The DNA cocktail labels simultaneously the lineage of any progenitors (green) and Dbx1-expressing progenitors (red). The location of labeled cells is analyzed 3 days post-electroporation. The labeled Dbx1-expressing progenitors produced a radial neuronal lineage at all pallial areas studied, including hippocampal anlage **(B)**, cingulate cortex **(E,F,I)** dorsal cortex **(G)**, lateral cortex and caudal cortex **(K,M,N)**. **(O,P)** Percentage of Dbx1-derived cells from the total of labeled cells across embryonic stages and cortical regions (rostro-lateral and caudo-medial cortex). Quantifications of Dbx1-derived remaining progenitors **(O)** and Dbx1-derived CP neurons **(P)** 3 days after electroporation and at rostro-lateral (RL) or Caudo-medial (CM) cortical levels. Dbx1-derived neurons represented a small, but significant fraction of the total electroporated cells. Error bars represent standard error of the mean. Scale bars represent 500 μm; 200 μm; and 100 μm in **(B–N)**.

Previous genetic fate-mapping experiments clearly show neurons within the CP generated from Dbx1-expressing progenitors (Teissier et al., 2010, 2012). We did not observe Dbx1-derived neurons migrating to dorsal cortex from the VP. In order to reconcile our results, we hypothesized that Dbx1-expressing progenitors might not only exist in the mouse VP, but might be sparsely present in other pallial areas as well, although this was not detected in previous reporter mouse lines (Teissier et al., 2010). To test this possibility, we first generated a plasmid to express Cre-recombinase under the control of the Dbx-1 promoter (pDBX1-Cre). This construct contains a 3 kb sequence upstream of the murine *Dbx1* gene, which encompasses the forebrain Dbx1 *cis*-regulatory enhancer (Lu et al., 1996) followed by the Hsp60 minimal promoter. We then transfected mammalian pallial progenitors with a combination of pPB-CAG-EGFP, pDbx1-Cre, and pPB-CAG-STOP-mCherry (Figure 3A). Such electroporations should only lead to mCherry fluorescent protein expression in progenitors where the Dbx1 promoter is active and in their descendants (Figure 3A). The pPB-CAG-EGFP plasmid in the transfected DNA cocktail provides a control for the electroporation, as it labels all progenitors regardless of their *Dbx1* locus activity. Contrary to *in situ* hybridization (Bielle et al., 2005), but similarly to genetic fate-mapping (Teissier et al., 2010), our method allows us to identify cell populations even when these express very low levels of Dbx1. Cre-recombinase triggers constitutive labeling of the subsequent lineage independent of the original level of Dbx1 expression. The Dbx1-selective labeling cocktail was electroporated into various different telencephalic areas at E11 (Figure 3), E12, E13, and E14 (Figure 4), when the generation of the glutamatergic transient population peaks (Teissier et al., 2010). If the VP is the only region of the pallium where *Dbx1* mRNA is expressed (Bielle et al., 2005), we would expect to find no red cells when transfecting the DP cortical neuroepithelium. Surprisingly, we found mCherry-labeled neurons in all pallial areas and developmental stages tested 3 days after electroporation [Figures 3, 4; control electroporations without Cre expressing construct showed no leakage from these arrested labeling plasmids (Garcia-Moreno et al., 2014)]. Brains electroporated at E11 and analyzed 3 days later showed scattered red cells within the electroporated area at both medial and lateral levels and throughout the rostro-caudal axis (Figures 3B–G). Similarly, brains electroporated at E12, E13, and E14, and analyzed 3 days later (Figure 4A) also showed a fraction of mCherry-expressing neurons at different medio-lateral and rostro-caudal levels (Figures 4B–N). These results indicate the presence of scattered Dbx1+ progenitors in all experimental conditions and pallial regions, and not restricted to VP. Dbx1-derived cells were found at similar proportions at rostro-lateral and caudo-medial levels of the cortex and at the three developmental time points quantified (Figures 3O,P). In the GZs, Dbx1-derived remaining cells 3 days after electroporation ranged from 26.7 to 12.8% of the total electroporated cells (Figure 3O). Based on their morphology and location, these cells are mostly progenitors, but also multipolar pyramidal cells. We did not use specific markers of cell cycle (PH3) or SVZ progenitors, (Tbr2). In the postmitotic CP, Dbx1-derived neurons ranged from 22 to 10.8% of the total of electroporated neurons (Figure 3P). Furthermore, it was reassuring that the Dbx1-selective cocktail did not label any cells at the SP (Figures 3F,G).

The results of our electroporations in VP in embryonic mice disagree with the existence of VP-derived cells migrating toward the DP. However, labeling performed through pDbx1-Cre in DP implies that Dbx1 is expressed in a reduced subset of local DP cortical progenitors.

## Discussion

A variety of telencephalic regions have been proposed to contribute to the development of the NCx. These areas generate glutamatergic neurons at early stages that migrate tangentially to colonize the developing NCx. In this study we show that the VP neurons, including the Dbx1 expressing population, do not migrate tangentially to the NCx as previously thought. We present evidence that Dbx1-derived cortical neurons are locally born within the neocortical primordium, and this could explain why cortical neuronal numbers are reduced in Dbx1 KO. Our results therefore suggest that VP influence on cortical development is less considerable than previously suggested.

### The Mammalian VP Does Not Contribute to the Neocortical Pyramidal Population

Recent studies suggest that a population of tangentially migrating glutamatergic pyramidal neurons originates at the Dbx1+ VP GZ in mouse (Teissier et al., 2010, 2012). Those studies used Dbx1-reporter gene assay, which showed strong expression of *Dbx1* mRNA in the mouse VP, and subsequently a transient population of glutamatergic neurons with prevalence toward the lower layers of cerebral cortex. However, our experiments, using a broad, direct lineage labeling from VP progenitors, revealed a lack of tangentially oriented cells directed toward the adjacent NCx in mouse. We observed no single labeled cells in dorsal cortex from electroporation sites in VP from E11 to E13 studied from E13 to E18). Whereas previous genetic fate mapping specifically targets a reduced population of progenitors (Teissier et al., 2010), our widespread and extensive electroporations indiscriminately labeled all progenitors in the area. In comparison to fate mapping studies, our electroporation experiments are less specific as they label a broader population of cells; and should, therefore, reveal more tangentially migrating populations, which strengthens our conclusion.

In order to align our interpretations to previous results, we propose that *Dbx1*, the gene triggering the reporter expression in the fate-mapping assays, is expressed not only in VP progenitors but also in a reduced number of DP cortical progenitors at low levels. These sparse, but significant, cortical Dbx1 expressing cell populations could not be detected by previous *Dbx1 in situ* hybridization (Bielle et al., 2005) but could be revealed by other means such as our selective electroporation using the Dbx1-promoter or genetic fate-mapping (Teissier et al., 2010, 2012). Our experiments suggest that there is a group of Dbx1-derived cortical neurons that are radial derivatives of ventricular DP precursors in mammals. This also explains the experiments in which Dbx1+ cells were selectively ablated (Teissier et al., 2010). In such a paradigm, there was a reduction of 20% in neocortical cell numbers and this decrease can be attributed directly to the loss of Dbx1+ DP progenitors and their radial progeny. This is in line with our estimates for the Dbx1-derived neurons that ranged from 22 to 10.8% of the total of electroporated neurons.

Short-term lineage tracing at different stages and levels of the VP never labeled a dorsally migrating population of neurons (Garcia-Moreno et al., 2008; Ceci et al., 2012; Frade-Pérez et al., 2017). *In toto* culture of embryos ranging from E10.5 to E12.5 is a valuable technique to describe the early steps of cellular migrations. In those experiments, the cell tracker CFDE-SE was injected directly into the VP neuroepithelium. The locally labeled cells only migrated ventrally and caudally, following olfactory-associated trajectories, but in no case did neurons migrate dorsally toward the neocortical neuroepithelium. Even in slice culture conditions, when cells are forced to move in a single plane of tissue, Dbx1-VP neurons only migrated and colonized the basal telencephalon (Figure 5 in Hirata et al., 2009).

Other published data support our proposal of local expression of Dbx1 at low levels in the NCx. Recently, new images of the Dbx1-LacZ mutant show lacZ expression at midneurogenic stages in the DP apical GZs but not in any CP region (Figures 2B–E in Puelles et al., 2015b). It is unlikely that these lacZ+ cells are tangentially migrating neurons given that migration in such deep GZs has never been observed before [it follows either the SVZ surface or the marginal zone (MZ); Figure 4 in Teissier et al., 2010]. Other studies performed independent genetic fate-mapping based on the regional telencephalic activity of selective enhancers and did not describe the VP tangential migration (Visel et al., 2013; Pattabiraman et al., 2014). The fate mapping using enhancer 636, specifically active in LP and VP GZs, marks cells only in the ventrolateral portion of the cerebral cortex, including insular and piriform cortices in an equivalent fashion to our electroporations. But no cells derived from this LP/VP region were observed in the CP (Figures 4 and S2E in Pattabiraman et al., 2014). However, labeling via enhancer 643, whose early activity is restricted to the cortical hem, labeled all cortical hem derivatives in late embryonic development, including tangential C-Rs of the NCx (enhancer 643, Figures 2,6,S2F in Pattabiraman et al., 2014). These two sets of independent data further support our hypothesis for the local generation of Dbx1-derived neurons, and point to no tangential migration of Dbx1 positive glutamatergic neurons from VP to cortex. We propose tangential migration is a more restricted feature than previously thought (Garcia-Moreno et al., 2018).

In evolutionary terms, the lack of tangential migration from mammalian VP seems also more parsimonious. It has recently been described that avian VP derivatives populate the mantle in an exclusively radial fashion (Garcia-Moreno et al., 2018), which we consider conserved across amniote species. However, the subplate and Cajal-Retzius tangential migrations are a mammalian-specific feature (Garcia-Moreno et al., 2018).

We need to consider the mouse strains employed in each study for possible differences. It has already been described that the lineage of selected cortical progenitors may vary on different genetic backgrounds of mouse strains (Gil-Sanz et al., 2015). Although it is unlikely that strain differences would account for such considerable differences in the origin of Dbx1-derived neurons in the mouse CP, these issues will need further investigation in various mouse strains and other mammalian species.

As a complementary hypothesis, the Dbx1-derived GABA and glutamatergic populations might have different origin. Dbx1-derived GABAergic cortical neurons could originate at a more distant telencephalic area, such as the preoptic area (PO) (Hirata et al., 2009; Gelman et al., 2011). According to this hypothesis the dual nature of Dbx1-derived neurons in the CP would reflect their multiple origin sites. Dbx1-derived glutamatergic neurons could arrive to the CP by radial migration from local cortical progenitors, as shown in our study, whereas, Dbx1-derived GABAergic neurons invade the NCx through tangential migration from the PO (Gelman et al., 2011). The more detailed analysis of these issues will have great implications to our current understanding of the developmental formation and evolutionary origin of the mammalian cerebral cortex.

## Author Contributions

FG-M and ZM contributed to conceptualization. ER-A, FG-M, and IM-G contributed to methodology and investigation. JME and ZM contributed to resources and funding acquisition. FG-M contributed to writing – original draft and visualization. FG-M and ZM supervised the data. All the authors wrote – reviewed and edited the manuscript.

## Conflict of Interest Statement

The authors declare that the research was conducted in the absence of any commercial or financial relationships that could be construed as a potential conflict of interest.

## Abbreviations

ac: anterior commissure
Ag: amygdala
Cl: claustrum
Cng: cingular cortex
CP: cortical plate
DP: dorsal pallium
GZ: germinative zone
Hc: hippocampus
HT: hypothalamus
Ins: insular cortex
IZ: intermediate zone
L5: cortical layer 5
L6: cortical layer 6
LP: lateral pallium
LV: lateral ventricle
MZ: marginal zone
NCx: neocortex
Pir: piriform cortex
PO: preoptic area
Se: septum
SP: subpallium
St: striatum
SVZ: subventricular zone
Th: thalamus
VMS: ventral migratory stream
VP: ventral pallium
